# A triad of double aortic arch, bicuspid valve, and aortic dilation: treat the right lesion!

**DOI:** 10.1093/ehjcr/ytae156

**Published:** 2024-03-26

**Authors:** Fabienne Dirbach, Christopher Roy, Matthias Stuber, Tobias Rutz

**Affiliations:** Service of Cardiology, Lausanne University Hospital and University of Lausanne, Rue du Bugnon 46, 1011 Lausanne, Vaud, Switzerland; Department of Radiology, Lausanne University Hospital and University of Lausanne, Lausanne, Switzerland; Department of Radiology, Lausanne University Hospital and University of Lausanne, Lausanne, Switzerland; Service of Cardiology, Lausanne University Hospital and University of Lausanne, Rue du Bugnon 46, 1011 Lausanne, Vaud, Switzerland

**Figure:**
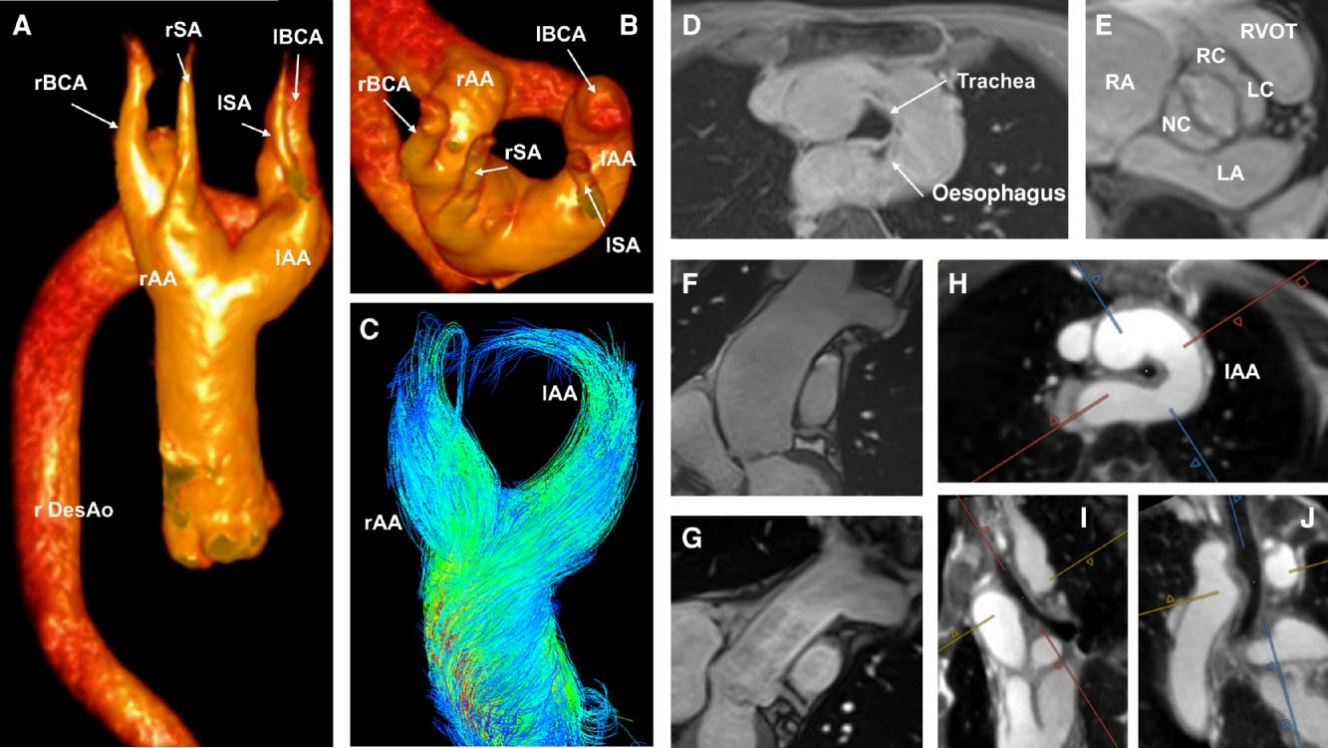
(*A* and *B*) Three-dimensional volume rendering of the free-running cardiac and respiratory motion-resolved 5D whole heart. (*C*) Four-dimensional flow cardiac magnetic resonance of both aortic arches. (*D*) Volumetric interpolated breath-hold examination (VIBE) showing the compressed trachea and oesophagus pre-operatively. (*E*) Still frame of 2D steady-state free precession (SSFP) cine of the bicuspid aortic valve. (*F*) Dilated ascending aorta before replacement. (*G*) Twenty-eight mm Gelweave tube in the position of the ascending aorta. (*H–J*) Multiplanar reconstruction in axial (*H*), sagittal (*I*), and coronal (*J*) planes of the free-running cardiac and respiratory motion-resolved 5D whole-heart images showing the compression of the trachea. LA, left atrium; lAA, left aortic arch; lBCA/rBCA, left/right brachiocephalic artery; LC, left coronary cusp; lSA, left subclavian artery; NC, non-coronary cusp; RA, right atrium; rAA, right aortic arch; RC, right coronary cusp; rDesAo, right descending aorta; rSA, right subclavian artery; RVOT, right ventricular outflow tract.

In a 71-year-old, healthy patient, an enlarged mediastinum was noticed on a chest X-ray performed for persistent cough after a respiratory infection. Computed tomography (CT) revealed a double aortic arch (DAA), a congenital heart lesion deriving from non-involution of the fourth aortic arches. Cardiac magnetic resonance (CMR) with 4D flow was performed showing the DAA with relatively balanced aortic arches (flow left arch, net 20 mL/heartbeat; right arch, net 15 mL/heartbeat; *Panels A* to *C*; Supplementary material online, *Movies S1* and *S2*), a tracheo-oesophageal compression (*Panel D*), and a fusion of both arches into a right descending aorta. Although the images suggested a highly symptomatic vascular ring, typically presenting with stridor, barky cough, dyspnoea, or dysphagia, the patient had never presented any of those symptoms. Cardiac magnetic resonance revealed further a bicuspid aortic valve with dilation of the ascending aorta of 52 mm (*Panels E* and *F*).

The heart team decided to surgically replace the ascending aorta at a supra-coronary level with a 28 mm Gelweave tube (*Panel G*) as the patient fulfilled the indications according to the European Society of Cardiology guidelines due to concomitant arterial hypertension. Owing to the absence of symptoms, the age and the balanced two aortic arches, the DAA, and the bicuspid valve, only showing mild insufficiency, remained untouched.

The post-operative course was uneventful. The patient remained asymptomatic to the DAA, although a free-running cardiac and respiratory motion-resolved 5D whole-heart suggested persistent symptomatic tracheo-oesophageal compression (*Panels H*–*J*; Supplementary material online, *Movies S3* and *S4*). Four-dimensional flow revealed a now completely balanced DAA (15 mL/heartbeat/branch each). Due to the concomitant absence of respiratory and swallowing symptoms and a normal clinical examination, no further investigations were undertaken.

This case highlights the role of advanced CMR techniques in addition to clinical evaluation to choose the right lesion requiring treatment.

## Supplementary Material

ytae156_Supplementary_Data

## Data Availability

The original data are available by the authors upon reasonable request.

